# The RAGE/DIAPH1 Signaling Axis & Implications for the Pathogenesis of Diabetic Complications

**DOI:** 10.3390/ijms23094579

**Published:** 2022-04-21

**Authors:** Ravichandran Ramasamy, Alexander Shekhtman, Ann Marie Schmidt

**Affiliations:** 1 Diabetes Research Program, Department of Medicine, New York University Grossman School of Medicine, New York, NY 10016, USA; ravichandran.ramasamy@nyulangone.org; 2 Department of Chemistry, The State University of New York at Albany, Albany, NY 12222, USA; ashekhtman@albany.edu

**Keywords:** RAGE, DIAPH1, interferon pathway, diabetes, diabetic complications, small molecule antagonist, diabetic kidney disease, diabetic accelerated atherosclerosis

## Abstract

Increasing evidence links the RAGE (receptor for advanced glycation end products)/DIAPH1 (Diaphanous 1) signaling axis to the pathogenesis of diabetic complications. RAGE is a multi-ligand receptor and through these ligand–receptor interactions, extensive maladaptive effects are exerted on cell types and tissues targeted for dysfunction in hyperglycemia observed in both type 1 and type 2 diabetes. Recent evidence indicates that RAGE ligands, acting as damage-associated molecular patterns molecules, or DAMPs, through RAGE may impact interferon signaling pathways, specifically through upregulation of IRF7 (interferon regulatory factor 7), thereby heralding and evoking pro-inflammatory effects on vulnerable tissues. Although successful targeting of RAGE in the clinical milieu has, to date, not been met with success, recent approaches to target RAGE intracellular signaling may hold promise to fill this critical gap. This review focuses on recent examples of highlights and updates to the pathobiology of RAGE and DIAPH1 in diabetic complications.

## 1. Introduction

Despite substantial public health efforts to combat obesity and type 2 diabetes (T2D), the epidemics of these common metabolic disorders continue undeterred [1,2]. Diabetes complications are major causes of morbidity and mortality, and often lead to significant loss of quality of life in affected persons as well as being a substantial financial burden to healthcare systems [1,2]. Many factors have been proposed as mediators of diabetic complications and one of the chief risk factors for diabetes is obesity. The underlying mediating factors in diabetic complications are reflected by the chemistry of the glucose molecule itself. Beyond the direct pathobiological effects of high concentrations of glucose in the diabetic organism, such as activation of protein kinase C isoforms, flux through the polyol pathway, hexosamine pathway flux, and oxidative stress [3], high concentrations of glucose trigger the Maillard reaction, in which an amino group of a protein/amino acid interacts with a reducing sugar [4]. Once this reaction is set in motion, a potential outcome is the generation of the irreversibly formed advanced glycation end-products, or AGEs.

As insights into the RAGE/DIAPH1 signaling axis continue to emerge, the opportunities to interrupt this pathological signaling pathway become more tangible. This review focuses on recent updates in the biology of RAGE and its cytoplasmic domain binding partner, DIAPH1 (Diaphanous 1) and provides updates to targeting this pathway.

### 1.1. Advanced Glycation End-Products (AGEs)

Spotlight on contributory roles for the AGEs in the pathogenesis of obesity and diabetic complications continues to grow [5]. Beyond the higher concentrations of the heterogenous groups of endogenously formed AGEs driven by hyperglycemia [6], exogenously derived AGEs, such as those imbibed through food, may play adjunctive roles in the pathogenesis of metabolic dysfunction [7]. Many of the endogenously generated AGE forms have also been detected in foods, such as the common and prevalent specific AGE, peptides and proteins modified by carboxymethyllysineAGE (CML-AGE), which are known ligands for the receptor for advanced glycation end-products (RAGE) [8,9]. Of note, evidence from structural biology studies suggests that free CML is not able to bind to RAGE, particularly its V-domain [10,11]. However, a distinct study in cultured cells using anti-RAGE IgG suggested that free CML might signal via RAGE; however, in that work, no direct structural biology studies were performed to directly test binding of free CML to RAGE [12]. Hence, more work is required to fully identify the minimal CML-related structure capable of binding to, and signaling, via RAGE.

Despite these considerations, the extent to which these food-derived AGEs are absorbed in their intact signal transduction-enabled forms, after gastrointestinal tract exposure to gastric acids and specific gut microbiota, as well as the mechanisms mediating absorption of these species across the gut, are yet to be fully clarified [6,13]. Furthermore, studies on the metabolic effects of high- vs. low-AGE diets are not conclusive. For example, studies in human participants with obesity revealed that one year of oral AGE restriction resulted in reduced insulin resistance compared to individuals consuming a regular AGE diet [14]. In contrast, a recent study reported that 4 weeks of consumption of a high-AGE diet in participants with obesity had no effect on glucose metabolism or vascular function [15]. The basis of these discrepancies may be due to such factors as the differences in the duration of AGE feeding, baseline adiposity, the metabolic state of the studies’ participants, and the specific foods and their preparation modes deemed as “high” vs. “regular” or “low”-AGE. Nevertheless, it is probable that solely targeting AGEs in obesity and diabetes therapy may not yield comprehensive protection from diabetic complications. In this context, there is considerable evidence that AGEs may bind to cellular receptors, thereby stimulating an array of cellular perturbations that cause tissue damage.

### 1.2. The Broad Swath of AGE Receptors

Given the heterogenous nature of AGEs, it is not surprising that there are a number of binding partners/receptors that have been identified for this class of molecules. The AGE receptors fall into two major groups, based on functional properties. In the first group, receptors such as RAGE, oligosaccharyl-transferase complex protein 48 (AGER1), 80 K-H protein (AGER2), and galectin-3 (AGER3) have been shown to bind AGEs and, through this binding, modulation of cellular properties ensues through various molecular modes [16,17,18,19,20]. In contrast, a distinct functional group of molecules serve as scavenger receptors (SR); among these are molecules such as class A type I and II (SR-AI/II), class B type I (SR-BI), CD36 and toll-like receptors (TLRs) [21,22,23,24]. In some cases, AGEs recruit the actions of multiple AGE receptors, such as RAGE and TLRs, in which one possible outcome is amplification of the AGE signal transduction consequences in the cells [25,26,27]. As RAGE is one of the best-characterized receptors for AGEs, this review focuses on the biology of RAGE in metabolic dysfunction and diabetes.

### 1.3. RAGE Is a Multi-Ligand Receptor

A critical piece of the complex biology of the RAGE molecule was uncovered through the discoveries that RAGE bound non-AGE ligands. The appended Table 1 summarizes the major ligands/ligand families of RAGE. As a member of the immunoglobulin (Ig) superfamily, the receptor is composed of multiple (three) and complex extracellular Ig-like domains. The extracellular Variable (V)-type Ig domain is followed by two Constant (C)-type Ig domains; there is some evidence that in certain cases, V-C1 operates as a functional unit. Whereas most of the primary classes of RAGE ligands, AGEs; amphoterin, also known as high mobility group box 1 (HMGB1); multiple members of the S100/calgranulin families; and amyloid-beta peptide bind to the V-type Ig domain, some of the ligands of RAGE may (also) bind outside of the V1 Ig-like domain onto the C1 or C2 Ig-like domains, such as S100A6, thereby adding complexity to the overall structure and functions of the RAGE pathway [28]. It is for this reason that small molecules or antibodies that target discrete sites on the extracellular V type Ig domain may not be fully effective on account of the ability of RAGE ligands to occupy distinct sites on the extracellular domains. For example, Azeliragon, which targets the V type Ig domain, ultimately failed to show primary endpoint efficacy in a Phase III clinical trial in Alzheimer’s disease [29]. These considerations led to the postulate that targeting the intracellular domain of RAGE might be a superior approach for the development of therapeutic approaches.

### 1.4. Diaphanous 1 (DIAPH1) Binds to the Cytoplasmic Tail of RAGE: Studies in Cell-Based Studies and In Vivo Models

To address the possibility that the cytoplasmic domain interacted with key effector molecules to recruit signal transduction pathways, a yeast two-hybrid screen was employed to identify binding partners of the RAGE cytoplasmic tail (ctRAGE). These experiments led to the discovery that the formin homology 1 (FH1) of Diaphanous 1 or DIAPH1 bound to ctRAGE [42]. Co-immunoprecipitation studies in cellular models verified this interaction; at the functional level, silencing of *Diaph1* expression in cells blocked RAGE ligand-mediated activation of the Rho GTPases, RAC1 and CDC42 and blocked the effects of RAGE ligands on cellular migration [42]. Other studies illustrated the effects of blocking DIAPH1 actions in RAGE ligand-stimulated signaling in smooth muscle cells, endothelial cells, adipocytes and cardiomyocytes [43,44,45,46]. In addition, experiments in mice globally devoid of *Diaph1* illustrated protective benefits in models of arterial injury, cardiac ischemia/reperfusion injury and diabetic kidney disease (DKD) [44,46,47].

### 1.5. ctRAGE-DIAPH1 Interactions and Structural Biology

Studies employing NMR spectroscopy techniques identified that ctRAGE contains an amino (N)-terminal segment that folds into an α-turn and a long unstructured carboxy (C)-terminal tail. It is established that α-turns typically contain mainly hydrophilic amino acids [48] and, accordingly, the α-turn of ctRAGE was found to consist of four charged amino acids, Arg-5, Arg-7, Arg-8, and Glu-10. Further experiments revealed that the FH1-ctRAGE interaction surface is located within the α-turn. Consistent with the importance of the amino acids R5 and Q6 in ctRAGE mediating the interaction with DIAPH1, mutation of these residues to alanine (R5A and Q6A) abolished the FH1–ctRAGE interaction by NMR spectroscopy. In cellular models, introduction of this double mutation blocked RAGE ligand-mediated signal transduction, migration and proliferation in cultured smooth muscle cells [49].

**Figure 1:**
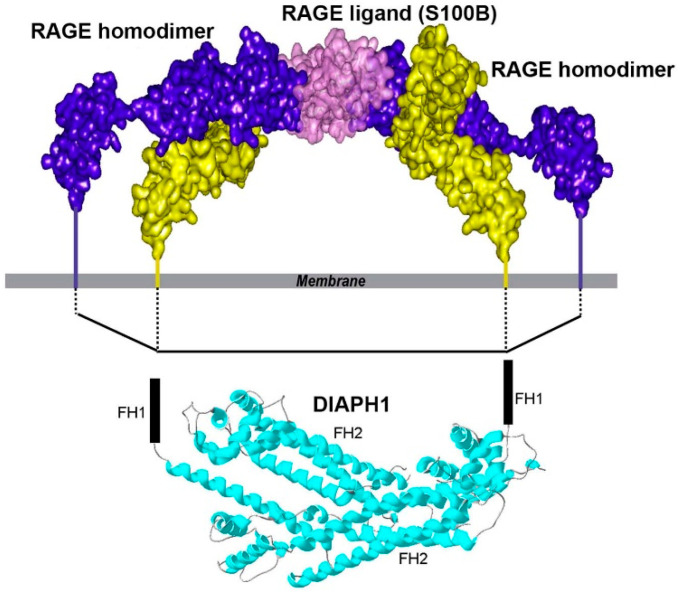
Ligand-dependent RAGE oligomerization facilitates binding of the intracellular domain of RAGE to the FH1-FH2 domains of DIAPH1. RAGE homodimers are illustrated in purple and yellow, respectively, and RAGE ligand (S100B) is in pink. Adapted from Reference [11], Xue et al. (Figure 5).

Further in-depth efforts to characterize RAGE-DIAPH1 interactions were spurred by the testing of two independent units of the extracellular domains—that is, the VC1 and C2 domains. From these studies, Shekhtman and colleagues crafted a model in which the negatively charged C2 Ig-like domain of RAGE stabilizes the soluble (s) RAGE homodimer structure by interacting with the positively charged VC1 domain, thereby reinforcing the importance of the VC1 and C2 domains for homodimerization of RAGE [11]. Using a variety of techniques, this team showed that ligand binding to the RAGE homodimers on the cell surface results in the recruitment of DIAPH1 and the consequent activation of signal transduction pathways [11]. Figure 1 summarizes the proposed RAGE/DIAPH1 model.

In distinct work, Smith and colleagues used super-resolution stochastic optical reconstruction microscopy (STORM) and single particle tracking (SPT) to study the effects of DIAPH1 in regulation of RAGE diffusion properties on the cellular surface. RAGE diffusion has been linked to actin cytoskeleton dynamics. In cultured cells bearing either mutation of R5/Q6 to alanine residues or in cells expressing RAGE but with marked reduction in DIAPH1 expression, the number and size of RAGE clusters was decreased compared to the respective control experiments [50]. The results of that work illustrated that DIAPH1 significantly affects RAGE clusters and diffusion. Importantly, these studies by Smith and her team reinforced the results of multiple distinct reports linking RAGE signaling to DIAPH1.

In the sections to follow, this review considers the evidence linking RAGE and DIAPH1 to well-known complications of diabetes—that is, atherosclerosis and diabetes-associated kidney diseases (DKD)—and presents the results of recent studies.

### 1.6. RAGE/DIAPH1 and Diabetic Atherosclerosis

Earlier studies suggested that pharmacological antagonism of the ligand–RAGE axis, using soluble RAGE, or genetic modifications such as global deletion of *Ager* (the gene encoding RAGE), attenuated acceleration of atherosclerosis progression in diabetic mice [51,52,53]. Studies using bone marrow transplantation strategies suggested that the contribution of RAGE to accelerated diabetic atherosclerosis ensued from both bone marrow- and non-bone marrow-derived mechanisms [54]. In the sections to follow, recent work highlighting new insights into RAGE/DIAPH1 and atherosclerosis are detailed.

#### 1.6.1. Micro-RNA (miR-21-3p) and RAGE

Recent studies have implicated microRNAs (miRs) in the regulation of production of the cleaved form of sRAGE. Exposure of cultured vascular endothelial cells to diabetes-relevant concentrations of glucose was found to decrease the expression levels of miR-21-3p and the introduction of miR-21-3p mimic significantly increased the expression of A Disintegrin and metalloproteinase domain-containing protein 10 (ADAM10) in these cells and, accordingly, in the presence of the miR-21-3p mimic, the concentrations of sRAGE were found to increase in these cells. In vivo, intravenous injections of miR-21-3p reduced diabetic atherosclerosis and increased serum levels of sRAGE in mice models, thereby implicating this miR in the regulation of RAGE ectodomain shedding [55]. These findings provide further insights into the mechanisms by which soluble RAGE (cleaved) may be produced in vivo and, thereby, highlight new therapeutic opportunities for diabetic atherosclerosis.

#### 1.6.2. Diabetes and Microcalcification: New Insights into Roles for Macrophages and Extracellular Vesicles

Recent studies have implicated RAGE in the production of extracellular vesicles with calcific potential, particularly in diabetes. Incubation of human primary macrophages with diabetes-relevant concentrations of glucose resulted in increased secretion of RAGE ligand, S100A9 and upregulation of the expression of RAGE protein in these cells. Treatment of human macrophages with S100A9 resulted in the increased expression of osteogenic and proinflammatory factors [56]. In addition, it was shown that the production of extracellular vesicles with calcific potential (as noted by alkaline phosphatase activity) was higher in the presence of recombinant S100A9 in human macrophages [56]. These responses in macrophages were traced to RAGE-dependent mechanisms, as silencing of either *S100A9* or *AGER* reduced these effects. These concepts were further tested in vivo in *Apoe* null mice rendered diabetic with streptozotocin; in these animals, treatment with *S100a9* siRNAs in macrophage-targeted lipid nanoparticles resulted in reduced microcalcification in the atherosclerotic plaques [56]. Experiments in human diabetic carotid artery plaques revealed that the expression of S100A9-RAGE was associated with markers of osteogenic activity and microcalcifications [56].

#### 1.6.3. Diabetes and Regression of Atherosclerosis

Despite lipid-lowering therapies, patients with diabetes experience fewer benefits with respect to the regression of established atherosclerosis compared to non-diabetic patients [57,58]. Remarkably, studies in mouse models of diabetes have recapitulated these key findings in that diabetes impairs atherosclerosis regression, as evidenced by the impaired reduction of plaque macrophages that typically ensues after lipid-lowering strategies [59,60,61,62]. In addition to impaired reduction in the content of plaque macrophages, diabetes has also been shown to sustain a proinflammatory response in these cells as well during regression [63]. In other studies, administration of Apolipoprotein A-1 to diabetic mice accelerated regression of atherosclerosis through the reduction of myelopoiesis and plaque inflammation [64]. Recent work has implicated RAGE and DIAPH1 in the mechanisms linked to impaired regression of diabetic atherosclerosis.

To test putative roles for RAGE/DIAPH1 in impaired regression of diabetic atherosclerosis, a murine aorta transplantation model was employed. In that model, transplantation of aortic arches from diabetic, hyperlipidemic Western diet-fed *Ldlr* null mice into diabetic normolipidemic recipient *Ager* null mice versus wild-type diabetic recipient mice accelerated regression of atherosclerosis. In addition to a significantly lower lesion area, lesion macrophage content, AGE content and oxidative stress were significantly lower in the diabetic recipient mice devoid of *Ager* vs. the wild-type recipient mice [65].

A key question from these studies was if/how RAGE affected macrophage properties. To address this point, donor mice devoid of the *Ldlr* that were homozygous for CD45.2 were used; in contrast, the recipient wild-type or *Ager* null mice were homozygous for CD45.1 By this strategy, after the transplantation, the effects of donor (*Ager*-expressing) vs. recipient (*Ager*-expressing or *Ager* null) macrophages could be dissected. RNA sequencing was performed on the CD45.2- vs. CD45.1-expressing macrophages retrieved from the regressing atherosclerotic plaques. These experiments illustrated the novel finding that RAGE-dependent mechanisms emanated principally from the recipient macrophages and linked RAGE to interferon signaling. RNA sequencing experiments revealed that deletion of *Ager* in the regressing plaques was associated with decreased macrophage expression of interferon regulatory factor 7 (*Irf7*). In bone marrow-derived macrophages (BMDMs) retrieved from wild-type and *Ager* null mice, it was reported that RAGE ligands upregulated expression of *Irf7* [65]. Silencing of *Irf7* expression in wild-type murine BMDMs resulted in a switch from pro- to anti-inflammatory gene expression in the presence of a cholesterol-rich environment. Furthermore, IRF7 regulated the expression of a number of genes linked to cholesterol metabolism. Similar to findings in diabetic murine atherosclerotic plaques, immunohistochemistry experiments colocalized IRF7 and macrophages in human atherosclerotic plaques [65]. While much work needs to be done to expand on these findings, this work nevertheless pointed to new roles for the ligand–RAGE axis in macrophage inflammation and cholesterol metabolism, at least in part through IRF7.

It is important to note that in the above-referenced study, aortic arches from diabetic mice devoid of the *Ldlr* were also transplanted into diabetic wild-type or *Diaph1* null mice [65]; compared to wild-type diabetic recipient mice, deletion of recipient *Diaph1* significantly accelerated regression of atherosclerosis, as lesion area and lesional macrophage content were significantly reduced by *Diaph1* deletion in the recipient mice. Notable in that work was the observation that plasma cholesterol levels in the diabetic standard chow-fed *Diaph1* null recipient mice were significantly lower than those levels observed in the diabetic wild-type mice [65]. Studies are underway to identify the mechanisms underlying these findings.

In the sections to follow, the role of RAGE/DIAPH1 in a key microvascular complication of diabetes—that is, in diabetic kidney disease (DKD)—is considered.

### 1.7. RAGE/DIAPH1 and Diabetic Kidney Disease

Multiple studies identified upregulated RAGE expression in the diabetic vs. non-diabetic human kidney, but also in non-diabetic kidney disease as well, such as in hypertension and autoimmune kidney abnormalities [66]. In that work, although RAGE expression was largely pinpointed in podocytes, expression of the receptor was noted in non-podocytes as well, such as in endothelial cells [66]. For the past two decades, multiple studies using gain-of-function genetic approaches [67], loss-of-function genetic approaches [68,69,70] and pharmacological approaches, such as sRAGE, anti-RAGE antibodies, DNA aptamers against AGEs or RAGE, and low-molecular weight heparin [68,69,71,72,73,74] were employed to illustrate roles for RAGE in murine models of type 1 and type 2 diabetic kidney disease (DKD). Interestingly, in a murine model of type 1 DKD using streptozotocin, roles for bone marrow-derived RAGE in the recruitment of immune cells and tubulointerstitial disease were illustrated [75].

The role of DIAPH1 in murine DKD was recently illustrated. Akin to findings regarding RAGE expression in DKD, it was shown that DIAPH1 was expressed in the human and murine diabetic kidney, at least in part in the tubulointerstitium and podocytes. *Diaph1* null and wild-type mice were rendered T1D-like with streptozotocin. After 6 months, compared with diabetic wild-type control animals, diabetic *Diaph1* null mice displayed significantly less mesangial sclerosis, podocyte effacement, glomerular basement thickening, and urinary albumin excretion [47]. Examination of the kidney cortex revealed that deletion of *Diaph1* in diabetic mice significantly reduced expression of genes linked to fibrosis and inflammation compared to controls [47].

In the sections to follow, recent studies reporting on RAGE/DIAPH1 in the diabetic kidney and findings relevant to tracking the AGE/RAGE axis in human patients with DKD are highlighted.

#### 1.7.1. HMGB1 and DKD: Studies in Mouse Models

As noted earlier, one of the most defining characteristics of the RAGE pathway is the ability of the extracellular domains of RAGE to bind non-AGE ligands. In this context, amphoterin or HMGB1 binds RAGE as well as other receptor families, such as the TLRs [76], and some studies suggest that, at least in certain milieus, RAGE and TLRs may act cooperatively, such as in the activation of downstream NF-kB [77]. Studies were performed in streptozotocin-induced diabetic mice in which the HMGB1 specific antagonist, called HMGB1 A box, was administered. Compared to vehicle (saline)-treated animals, BALB/c mice treated with the HMGB1 A box protected against pathological and functional indices of DKD [78]. Of note, in these mice, the treatment with HMGB1 A box had no effect on body weight or on the degree of hyperglycemia. Mechanistically, although the treatment with the inhibitor of HMGB1 had no effect on expression of *Tnf* mRNA in the kidney, transcripts for multiple markers of fibrosis and inflammation (*Tgfb*, *Cxcl10*, *Ccl2*, and *Fn1*) were significantly attenuated by treatment with HMGB1 A box [78].

These important findings add to the concept that RAGE-related diseases are very likely not accounted for by single ligands; rather, it is more likely that in the diabetic tissue environment with excess inflammation and oxidative stress, multiple ligands beyond AGEs contribute to the overall pathologies.

#### 1.7.2. Anti-RAGE Vaccination and a New Therapeutic Opportunity for DKD

In recent studies, a RAGE “vaccine” was generated through the preparation of RAGE peptide (amino acids 38–44) coupled to keyhole limpet hemocyanin (KLH). Mice with type 1 (streptozotocin) and type 2 diabetes (*db/db* mice) were immunized through three rounds of injections; for up to 38 weeks, the evidence of the immunization was apparent through analysis of anti-RAGE antibody titers [79]. It was notable that in both the type 1 and type 2 models, the administration of the vaccine resulted in significant attenuation of pathological and functional indices of DKD [79]. Although potential side effects were not a focus of the study, it is nevertheless intriguing that opportunities to disarm RAGE through vaccination may hold promise for future directions.

#### 1.7.3. Acute Glucose Fluctuation and Upregulation of RAGE

With the advent of continuous glucose monitoring (CGM), new insights are emerging regarding the pathological consequences of acute glucose fluctuations (AGF). Based on the recordings from continuous glucose monitoring, in persons with diabetes, higher time-in-range (TIR), that is, without dramatic fluctuations, was associated with reduced risks of albuminuria, retinopathy, cardiovascular all-cause mortality, all-cause mortality, and abnormal carotid intima-media thickness (IMT) [80]. Recent studies in cultured rat podocytes showed that 72 h treatment with AGF resulted in a number of pathological consequences in these cells, such as inhibition of proliferation, upregulation of inflammatory molecules such as TNF and IL-1beta, increased oxidative stress and autophagy. One of the consequences of AGF in these cells was the upregulation of RAGE, which resulted at least in part due to oxidative stress/NFKB-driven pathways [81]. Evidence for mechanistic roles for oxidative stress in AGF-mediated upregulation of RAGE was discerned from experiments in which pretreatment of the podocytes with two different antioxidants (*N*-acetyl cysteine or pyrrolidine dithiocarbamate) resulted in cellular protection [81].

Although these authors did not test these concepts in vivo with respect to DKD, their work nevertheless identifies a specific series of AGF–oxidative stress-driven cellular damage, at least in part through RAGE. In fact, in other studies, in atherosclerosis, it was shown that intermittent intraperitoneal boluses of glucose administration accelerated atherosclerosis in mice devoid of *Apoe* without exerting direct effect on plasma levels of cholesterol [82]. Mechanistically, transient intermittent hyperglycemia resulted in bone marrow myelopoiesis, which caused increased circulating monocytes, especially the inflammatory Ly6-C^hi^ subset, and neutrophils. The mechanisms were then traced to an S100A9-RAGE-dependent pathway, as increased glucose uptake in the white blood cells enhanced glycolysis and resulted in the production of S100A8/A9. These processes were suppressed by myeloid-specific deletion of *Slc2a1* (GLUT1), blockade of S100A8/9 or global deletion of *Ager* [82].

#### 1.7.4. Updates on Tracking sRAGEs in DKD

The measurement of concentrations of sRAGEs (total sRAGE, endogenous secretory (es)RAGE, cleaved sRAGE [=total sRAGE − esRAGE]) reveals variable findings in human subjects with various RAGE-related disorders; in some studies, “low” vs. “high” concentrations of these soluble forms have been found to be associated with better or worse DKD-related disease and the reasons for these apparent discrepancies have yet to be understood [83,84]. Based on this consideration, studies have begun to postulate that simultaneous measurement of sRAGE and RAGE ligand AGE levels may be a better predictor of renal function. One study reported that the ratios of AGEs/sRAGE, AGEs/cRAGE, and AGEs/esRAGE (all of these were measured by ELISA) were elevated in end-stage renal disease (ESRD) [84]. Others reported that the individual associations of AGEs, sRAGE or esRAGE (and their ratios) varied across the spectrum of time and disease burden [85].

It is important to note that in many of the reported studies, patients with and without diabetes were included in the same study; it remains possible that the added burden of hyperglycemia shifted the AGE/sRAGEs dynamic such that the (in)ability to excrete these factors was amplified on account of very high production. Nevertheless, the important recent observation that simultaneous measurement of AGEs and sRAGE isoforms may be superior to either factor alone in testing associations with renal dysfunction (and its potential progression) paves the way for prospective studies to address these important questions.

Finally, in the section to follow, updates on targeting the intracellular domain of RAGE for therapeutic modulation of RAGE signaling are presented.

#### 1.7.5. Small Molecules Targeting the Cytoplasmic Domain of RAGE Interaction with DIAPH1

Upon the discovery that the cytoplasmic domain of RAGE bound the FH1 domain of DIAPH1 and that mutations in the ctRAGE that bound DIAPH1 blocked RAGE ligand-stimulated signaling and functional properties in vascular cells [49], this finding was leveraged to test the possibility that small molecules binding to ctRAGE might block these pathological interactions. Accordingly, a >58,000 small molecule compound library was probed for molecules that blocked the interaction of the RAGE cytoplasmic domain with DIAPH1. From this screen, approximately 13 molecules were identified that met in vitro binding parameters as well as activities in cultured cells stimulated with RAGE ligands [86]. These 13 small molecules served as backbone structure probes for further structure–function chemistry modifications to enhance their potency, solubility and in vitro and in vivo pharmacologic properties.

From the 13 molecules, one of these, based on the 2-phenylquinoline scaffold, has been undergoing extensive further development. A recently identified novel chemical probe identified from this scaffold, called “RAGE229”, which is *N*-(4-(7-cyano-4-(morpholin-4ylmethyl)quinolin-2-yl)phenyl)acetamide, demonstrated significant efficacy in targeting RAGE-DIAPH1 in multiple assays [87]. In tryptophan fluorescence quenching assays, the affinity of RAGE229 for ct RAGE was K_D_ 2 ± 1 nM. NMR spectroscopy studies verified the interaction and illustrated the key amino acids in the RAGE tail to which RAGE229 bound. In Förster resonance energy transfer (FRET) experiments, treatment with RAGE229 was found to decrease the FRET signal between the cytoplasmic tail of RAGE and DIAPH1 in a dose-dependent manner [87].

In addition to in vitro binding studies, experiments in cultured cells (expressing RAGE and DIAPH1) illustrated the effects of RAGE229 on blocking RAGE ligand-stimulated cellular migration (after a scratch wound) in murine and human vascular smooth muscle cells and blocked RAGE ligand-stimulated signal transduction (phosphorylation of ERK and AKT) in these cells. Ex vivo experiments in diabetic mouse hearts retrieved from hyperglycemic Akita mice showed that RAGE229 reduced hypoxia/reperfusion injury when compared to vehicle treatments [87]. In vivo studies in streptozotocin-induced diabetic mice undergoing myocardial infarction revealed significantly lower infarct area and improved recovery of cardiac function in RAGE229- vs. vehicle-treated mice [87]. Collectively, these studies illustrated that RAGE229 was effective for the reduction of injuries in diabetic mice in short-term models of complications.

To address the need for identifying strategies to target long-term complications of diabetes, two distinct areas were addressed. In the first, male and female BTBR *ob/ob* mice underwent full-thickness excisional wounding and were treated topically from days 3–10 post-wounding with either RAGE229 or vehicle. Male but not female mice treated with RAGE229 displayed a significant improvement in wound closure; based on pathological analysis and scoring of the wounds for key indices of wound healing, both male and female mice treated with RAGE229 demonstrated significant improvements vs. those animals treated with vehicle [87].

In the second diabetic complications area, mice with type 1 and type 2-like DKD were treated with RAGE229 after the establishment of hyperglycemia. In the type 1-like DKD model, male and female mice with streptozotocin-induced hyperglycemia were treated daily with three different doses of RAGE229, to deliver 150 ppm/day, 50 ppm/day, or 15 ppm/day in medicated chow. In both sexes, the highest dose of RAGE229 in general provided the greatest protection against pathological (Periodic Acid Schiff and electron microscopy analyses) and functional (urine albumin excretion) endpoints [87]. Importantly, the benefits of RAGE229 treatment were independent of changes in the concentrations of blood glucose.

In the type 2-like DKD model, male and female BTBR *ob/ob* mice were treated with RAGE229 only at the highest dose, delivering 150 ppm of RAGE229/ay in medicated chow. In both sexes, RAGE229 exerted significant benefits against the above pathological and functional indices of DKD in mice. As in the case of the type 1-like DKD model, treatment with RAGE229 did not lower the concentrations of blood glucose in the diabetic mice, indicating that its benefits were independent of hyperglycemia [87].

In the section to follow, the effects of the chemical probe RAGE229 on inflammation and inflammatory biomarkers will be presented; the results of these studies hold promise for the identification of biomarkers to track the effects of RAGE229 in vivo.

#### 1.7.6. RAGE229 and Dampening the Inflammatory Response: Paving the Way to Identifying Biomarkers to Track RAGE-Dependent Inflammation In Vivo

One of the first in vivo settings in which the earliest antagonist of ligand-RAGE interaction, sRAGE), was tested was in a murine model of methylated bovine serum albumin (mBSA)-mediated delayed type hypersensitivity (DTH) in non-diabetic mice [31]. In the DTH study adapted to the study of RAGE229, mice were sensitized over the inguinal lymph nodes with mBSA. Three weeks later, four doses of RAGE229 were administered by oral gavage or by intraperitoneal treatment just prior to the foot pad challenge with mBSA, and then the inflammation score was determined 12 h later [87]; in other studies, mice were fed for 7 days continuously with RAGE229-medicated chow. In all of these experimental settings, irrespective of the route of administration, RAGE229 treatment significantly reduced foot pad inflammation score vs. vehicle [87]. These results raised the possibility that in the long-term diabetes models (type 1 and type 2-like DKD), treatment with RAGE229 might reduce diabetes-associated upregulation of inflammatory markers.

In the type 1-like DKD model, diabetes resulted in a significant increase in plasma TNF-α concentrations in both male and female mice. Whereas male mice receiving RAGE229-chow at 150, 50, or 15 ppm demonstrated reduced plasma TNF-α versus vehicle treatment, no effects were noted in the female diabetic mice treated with RAGE229. Other markers were also studied; in both male and female mice, plasma interleukin-6 (IL-6) was increased in vehicle-treated diabetic mice compared with nondiabetic mice and in both male and female mice, treatment with RAGE229 at 150, 50, or 15 ppm resulted in significantly lower IL-6 concentrations compared with vehicle-treated mice. Analogous findings were noted in the case of plasma C-C motif chemokine ligand 2 (CCL2)/JE monocyte chemoattractant peptide-1 (JE/MCP1), which was upregulated in diabetes and at 150 or 50 ppm/day, in both male and female mice, significantly lower CCL2 was noted vs. vehicle treatment [87].

These concepts were also tested in the type 2-DKD BTBR *ob/ob* mice. In both the male and female mice, diabetic BTBR *ob/ob* mice displayed significantly higher plasma TNF-α, IL6 and CCL2 concentrations compared with nondiabetic control mice. In both male and female BTBR *ob/ob* diabetic mice, treatment with RAGE229-containing chow (150 ppm) reduced plasma TNF-α, IL-6 and CCL2 concentrations compared with vehicle treatment [87]. These findings indicate that diabetes increases plasma concentrations of numerous inflammatory molecules in both type 1-like and type 2 murine diabetes models and that these elevations may be reduced by RAGE229, largely, but not always, in both sexes. It is anticipated that these inflammatory mediators, as well as others, may ultimately be tested in human patients with diabetes during early-phase clinical trials. In the broader context, the published evidence supports roles for RAGE/DIAPH1 in distinct complications of diabetes; these areas will be considered in the section to follow.

#### 1.7.7. RAGE/DIAPH1: Broad Implications for the Complications of Diabetes

As the products of nonenzymatic glycation and oxidation of proteins accumulate systemically, RAGE/DIAPH1 has been shown to contribute to a broad range of long-term complications. Although this review detailed roles for this axis in macrovascular atherosclerosis and microvascular diabetic kidney disease, RAGE/DIAPH1 has been shown to play damaging roles in other tissues vulnerable to complications as well.

In the diabetic heart, evidence links RAGE and its ligands to exacerbation of ischemic damage [88]. Interestingly, recent studies in human subjects examined circulating concentrations of RAGE ligands involved in cardiac complications (AGEs, S100B, S100A1, S100A6, and the pro-apoptotic Fas ligand (FasL)); the authors reported that in middle-aged patients with T2D, higher levels of circulating S100B, AGEs and FasL and lower levels of sRAGE, S100A1 and S100A6 were observed when compared to patients with normal or impaired glucose tolerance who did not have T2D [89]. These authors proposed the hypothesis that the lower levels of sRAGE may limit decoy and exogenous trapping of deleterious pro-apoptotic/pro-inflammatory ligands that have been shown to accumulate in and contribute to diabetic complications.

In other studies in human subjects with coronary artery disease, the relationship between the expression of RAGE and the thickness of epicardial adipose tissue (EAT) was examined. This work revealed a strong positive correlation between RAGE expression and EAT thickness [90]. Furthermore, it was reported that RAGE expression in EAT was associated with reduced expression of GLUT4 (glucose transporter type 4), adiponectin, and Glyoxalases 1 (GLO1), and elevations of HMGB1, TLR4, and MYD88 (myeloid differentiation primary response 88). The authors concluded by suggesting that RAGE may be involved in the promotion of EAT adiposity and consequent metabolic dysfunction.

In the diabetic retina, earlier studies in mice models employed treatment with sRAGE in diabetic (*db/db*) mice devoid of *Apoe* to illustrate beneficial effects on retinal vasculature and on electroretinograms (ERGs) [91]. Key studies were performed in STZ-induced diabetic mice expressing or devoid of *Ager.* In that work, diabetic mice devoid of *Ager* demonstrated significantly less vascular permeability, leukostasis and activation of microglia [92]. Although the mice devoid of *Ager* were protected from the formation of acellular capillaries in diabetes, there was no effect on pericyte loss [92]. Recent studies in non-human primates studied spontaneous diabetes in cynomolgus monkeys that were >15 years of age. Retinal pigment epithelial (RPE) cells were dissected from the retinas and subjected to RNA sequencing [93]. Among the major diabetes-associated pathways were the “AGE–RAGE signaling pathway” and pathways linked to inflammation, lipid metabolism, apoptosis and complement activation [93]. In parallel, plasma concentrations of C3 and C4 were found to be increased in the diabetic monkeys vs. age-matched control monkeys [93]. Of note, earlier work showed that C1q bound RAGE and that this interaction contributed to phagocytosis [37]. If and how RAGE and complement activation are linked to the pathogenesis of diabetic retinopathy remain to be explored.

In the diabetic nervous system, numerous studies have localized RAGE to diabetic nerves. In the skin of human subjects with diabetes, RAGE expression was upregulated, particularly in the dermal and subcutaneous vascular endothelium, and the extent of *AGER* mRNA transcript elevation in the diabetic skin was higher in severe vs. milder or no neuropathy [94]. Multiple studies examined the role of RAGE in animal models; for example, earlier studies showed that deletion of *Ager* improved pain perception in diabetic mice [95]. Recent studies illustrated roles for HMGB1 in painful diabetic neuropathy, as treatment of diabetic mice or rats with the HMGB1 inhibitor, glycyrrhizin, improved mechanical and thermal pain threshold [96].

Recent reviews have also covered roles for AGEs/RAGE ligands and RAGE in the pathogenesis of diabetic complications [17,97,98]. Targeting RAGE for the prevention/treatment of diabetic complications, as well as other RAGE-related disorders, is the critical requirement for the discovery of RAGE’s innate functions.

### 1.8. Perspectives on the Therapeutic Approaches to Targeting RAGE/DIAPH1 in the Clinic: What Are the Natural Functions of RAGE/DIAPH1?

Recent work has illustrated that deletion of *Ager*, globally or specifically in adipocytes, protected mice from high-fat diet-induced obesity. The underlying mechanisms were traced to RAGE ligand-RAGE-dependent suppression of protein kinase A (activities), consequences of which included reduced lipolysis and suppression of Uncoupling Protein 1 (UCP1). In parallel with reduced diet-induced obesity, mice devoid of *Ager* globally or in adipocytes displayed reduced insulin resistance [99,100]. These considerations suggested a plausible innate function for RAGE in the context of conservation of energy, which, along the course of evolution, might have imparted beneficial effects during times of scarce nutrients. However, during excess energy intake, the ligands of RAGE, such as CML–AGE, accumulate and may further contribute to suppression of energy expenditure. In this context, it is plausible that an auxiliary benefit of antagonism of RAGE may be a release from downregulated energy expenditure, and, therefore, an overall improvement in metabolic health.

**Figure 2:**
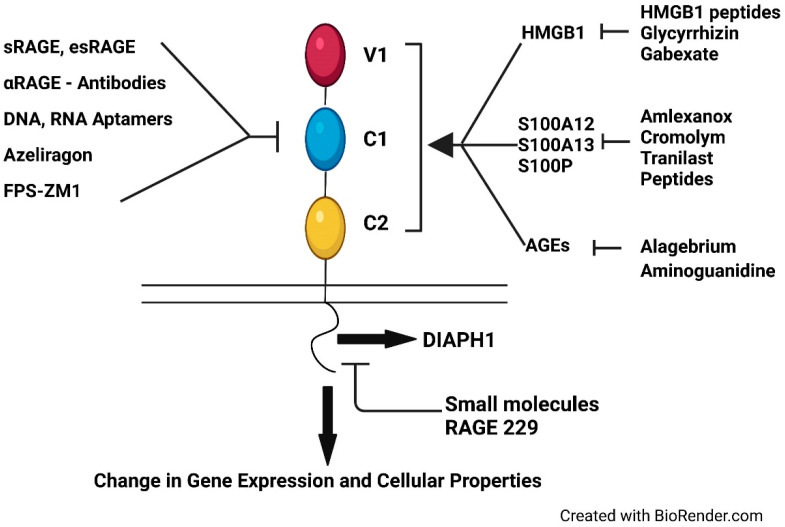
Examples of RAGE-directed therapies. Shown in the Figure (**left** side) are examples of strategies that target one or more of the RAGE extracellular domains; shown in the Figure (**right** side) are examples of strategies that target the ligands that may bind to RAGE. At Figure center, bottom, are depicted examples of strategies that target the interaction of the RAGE cytoplasmic domain with DIAPH1.

In this context, numerous approaches have been reported to target the RAGE signaling pathway and examples of these are depicted in Figure 2. From the very first means to target RAGE, that is, administration of soluble RAGE [101], to small molecule antagonists that uniquely target the RAGE cytoplasmic domain [86,87], thereby impeding its interaction with DIAPH1, RAGE and RAGE signaling remain an attractive therapeutic opportunity for disorders in which its ligands accumulate through strategies that target RAGE domain(s) or target specific ligands of RAGE; examples of these are reported in the following references [31,71,72,102,103,104,105,106,107,108,109,110,111,112,113]. Despite the potential benefits of RAGE antagonism on such disorders, an open question remains regarding the possibility of protective roles for the RAGE axis in discrete types of cells, such as immune cells. Although in a range of experiments testing the RAGE pathway in induced infections, including bacteria, viruses, and parasites [114,115,116,117] and, most recently, SARS-CoV-2 [118], salutary and non-detrimental effects were noted upon genetic *Ager* deletion or pharmacological antagonism of RAGE, these experiments were conducted under controlled conditions and most often in healthy, young mice. Thus, it remains to be seen if increased infection might be observed upon chronic RAGE antagonism in the more complex setting of aging and chronic disease in human subjects. However, it is important to note that up to 18 months treatment with azeliragon (small molecule RAGE antagonist targeting the extracellular domains) in older patients with cognitive impairment did not result in the reporting of increased susceptibility to infections or other infectious sequelae [29].

In conclusion, what, then, are the next steps in the evolving studies on RAGE, RAGE signal transduction, and the relationships to DIAPH1? Here, it is proposed that essential directions should complement extensive studies in mice globally devoid of *Ager* (or *Diaph1*) and administration of systemic antagonists of RAGE signaling with new strategies such as the production of *Ager-* and *Diaph1*-modified mice through bone marrow transplantation and the use of tissue-targeted mice with cell type-specific deletion of *Ager* or *Diaph1* to facilitate the discovery of the full range of RAGE- and RAGE/DIAPH1-dependent functions in innate immunity. It is speculated that at least in certain tissues/organs and in discrete types of cell stress, such methodologies may uncover innate functions for the receptor signaling axis in vivo. It is proposed that this knowledge is essential to fully outline the most logical settings for effective targeting of the RAGE/DIAPH1 signaling axis.

## Figures and Tables

**Table 1:** Examples of major ligands that bind to the extracellular domains of RAGE.

Ligand/Ligand Family	Reference
AGEs (advanced glycation end-products)	[30]
S100A12, S100B (+other S100/calgranulins)	[31]
HMGB1 (Amphoterin)	[32,33]
Amyloid beta-peptide	[34]
Islet amyloid polypeptide	[35]
Mac-1	[36]
C1q	[37]
Lysophosphatidic acid (LPA)	[38]
Phosphatidylserine (PS)	[39]
RNA, DNA	[40,41]
